# A Case of Anti-N-Methyl-D-Aspartate Receptor (NMDAR) Encephalitis With Video-Documented Psychogenic Nonepileptic Seizures (PNES)-Mimicking Episodes Initially Considered as Somatic Symptom Disorder

**DOI:** 10.7759/cureus.79384

**Published:** 2025-02-20

**Authors:** Ruoyi Ishikawa, Takamichi Sugimoto, Narumi Ohno, Takahiro Iizuka, Eiichi Nomura

**Affiliations:** 1 Department of Clinical Neuroscience and Therapeutics, Hiroshima University, Hiroshima, JPN; 2 Department of Neurology, Hiroshima City Hiroshima Citizens Hospital, Hiroshima, JPN; 3 Department of Clinical Neuroscience and Therapeutics, Hiroshima University Graduate School of Biomedical and Health Sciences, Hiroshima, JPN; 4 Department of Neurology, Kitasato University, School of Medicine, Kanagawa, JPN

**Keywords:** anti-nmda receptor encephalitis, autoimmune encephalitis, cell-based assay, cyclophosphamide pulse, extreme delta brush, immunotherapy, missed diagnosis, psychogenic nonepileptic seizures (pnes), somatic symptom disorders, video

## Abstract

Anti-N-methyl-D-aspartate receptor (NMDAR) encephalitis is an autoimmune disorder characterized by psychiatric symptoms, seizures, and dyskinesias. This case report describes a 30-year-old woman who was initially suspected of having a somatic symptom disorder because of the development of seizures mimicking psychogenic non-epileptic seizures (PNES). At presentation, she was able to engage in conversation and follow instructions, but exhibited slight fever, sensory abnormalities, and non-stereotypical seizures. Over the course of two weeks, she fell into a catatonic stupor. Cerebrospinal fluid (CSF) analysis revealed only mild pleocytosis with CSF-restricted oligoclonal bands. Electroencephalogram, which was unremarkable at presentation, subsequently showed an extreme delta brush pattern. NMDAR antibodies were detected in CSF with two independent assays, confirming the diagnosis of anti-NMDAR encephalitis. First-line immunotherapy with steroids, plasma exchange, and immunoglobulins was ineffective, but second-line immunotherapy with cyclophosphamide led to improvement. This case underscores the importance of considering anti-NMDAR encephalitis in patients with PNES-mimicking episodes, which can be misleading and delay appropriate diagnosis and treatment.

## Introduction

Anti-N-methyl-D-aspartate receptor (NMDAR) encephalitis is an autoimmune disorder characterized by psychiatric symptoms, seizures, speech dysfunction, involuntary movements, decreased level of consciousness, and central hypoventilation/autonomic dysfunction [1,2]. It is caused by autoantibodies against the NR1 subunits of the NMDAR (GluN1 antibodies), with an estimated annual incidence of approximately 1.5 per million people. It predominantly affects children and young adults, with a female predominance, as approximately 80% of cases occur in women, often in association with ovarian teratomas [2]. Racial and ethnic disparities have been observed, with higher incidence reported among Black, Hispanic, and Asian populations compared to White populations [3]. The diagnosis of anti-NMDAR encephalitis is commonly guided by the criteria proposed by Graus et al., which emphasize the rapid onset of psychiatric symptoms, seizures, movement disorders, and autonomic dysfunction, alongside supportive findings from cerebrospinal fluid (CSF) or electroencephalography (EEG) [4,5]. Early recognition and prompt immunotherapy are crucial for improving patient outcomes.

Diverse psychiatric symptoms have been described but this disorder typically begins with acute onset of progressive schizophrenia-like symptoms, such as hallucinations, delusions, anxiety, insomnia, and abnormal behavior [6,7]; however, not much attention has been paid to seizures mimicking psychogenic non-epileptic seizures (PNES). Here, we report a patient who was initially suspected of having a somatic symptom disorder due to PNES-mimicking episodes, which were later confirmed to be part of the phenotypic spectrum of anti-NMDAR encephalitis.

## Case presentation

A previously healthy 30-year-old Japanese woman was admitted to the Department of Neurology of our hospital in January 2020 with recurrent generalized trembling seizures. The patient had been in her usual state of health until approximately three weeks before this admission, when fever developed. The temperature spontaneously declined; however, an intermittent tingling sensation began in her lower limbs in late December 2019.

One day before this admission, she visited the outpatient clinic. She reported difficulty standing, but she was able to walk with assistance, and neurologic examination was unremarkable; however, she subsequently developed a seizure, beginning with unnatural trembling motions of her head and trunk followed by an opisthotonic posture while shaking her arms bilaterally with elbow extension, mimicking PNES (see Video 1). She developed similar trembling seizures, during which she exhibited a delayed response to questions, but she was able to follow commands and answer her name or date correctly.

**Video 1:** Psychogenic nonepileptic seizure (PNES)-mimicking episodes This video was recorded one day before admission. Note the paroxysmal movement disorder or seizure mimicking psychogenic non-epileptic seizures, which begins with unnatural trembling motions of her head and trunk, followed by an opisthotonic posture while shaking her arms bilaterally with elbow extension (see text).

**Figure 1:**
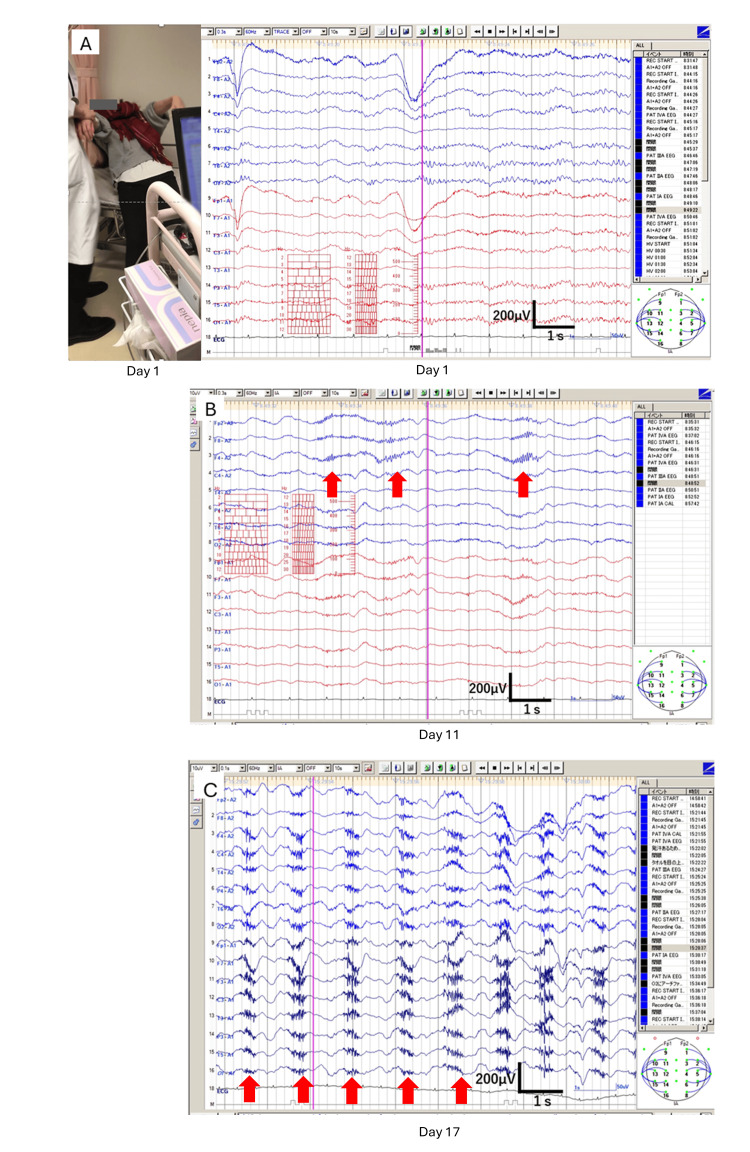
Electroencephalogram The electroencephalogram (EEG) recorded one day before admission was unremarkable (A). Mild extreme delta brush, predominantly in the frontal regions, appeared on day 11 of admission (B) and became more prominent on day 17 (C). The red arrows indicate the extreme delta brush. The EEG is a 10-second epoch, recorded using a monopolar montage with a sensitivity of 10 μV/mm and a gain of 10,000. The vertical scale bar represents 200 µV, and the horizontal scale bar represents 1 s.

An EEG was unremarkable (Figure 1A). Based on the phenotype of seizures, she was suspected of having a somatic symptom disorder, and was discharged home; however, her family, who discreetly observed her, witnessed multiple seizure attacks occurring even when she was alone in her room without an accompanying person. The following day, she was admitted to our hospital for further evaluation and treatment.

General physical findings

The temperature was 37.3°C, the blood pressure was 135/77 mm Hg, the pulse was 85 beats per minute, and the oxygen saturation was 97% while the patient was breathing ambient air, with a Glasgow Coma Scale (GCS) score of 14 (E4V4M6). Physical examination was otherwise unremarkable.

Neurological findings

On admission, the patient was awake and had no prominent psychiatric symptoms, but had tearful eyes and unstable emotions; she spoke spontaneously but did not make eye contact with the examiner. She partially responded to complex inquiries; for instance, when informed about a referral to psychiatry, she expressed a preference for a female psychiatrist. The function of the cranial nerves was intact. Strength was almost normal, but she had give-way weakness. Reflexes were mildly exaggerated at the knees and ankles, but plantar responses were flexor bilaterally. She reported dysesthesia on the dorsum of her feet, but sensory examination was objectively unremarkable, and the area of abnormal sensation was not consistent with peripheral nerve innervation. She had no appendicular ataxia. Although she was able to stand on one foot and walk with a tandem gait without assistance, she exhibited an unsteady gait and leaned on an object or a person while standing or walking. The neck was supple. During the examination, she did not develop trembling seizures.

Key laboratory findings

Laboratory assessment on admission was unremarkable, including complete blood count, the levels of electrolytes, C-reactive protein (CRP), and the results of tests of liver, renal, and thyroid function. Angiotensin-converting enzyme, antinuclear antibodies, myeloperoxidase-antineutrophil cytoplasmic antibodies, proteinase 3-antineutrophil cytoplasmic antibodies, and antibodies to double-stranded DNA (dsDNA), Sjögren’s-syndrome-related antigen A (SS-A/Ro), and Sjögren’s-syndrome-related antigen B (SS-B/La), glutamic acid decarboxylase, myelin oligodendrocyte glycoprotein, and aquaporin-4 were examined and were negative. However, thyroglobulin (Tg) antibodies (648 IU/mL; normal range: <28 IU/mL) and thyroid peroxidase (TPO) antibodies (116 IU/mL; normal range: <16 IU/mL) were elevated.

CSF examination obtained on day two of admission revealed 19 white blood cells (WBCs)/µL (100% mononuclear cells), a protein level of 29 mg/dL, a glucose level of 59 mg/dL, an IgG index of 0.69 (normal range: 0.3-0.7), and CSF-restricted oligoclonal bands (OCBs) were detected. Herpes simplex virus (HSV) polymerase chain reaction (PCR), cryptococcal antigen testing, India ink staining, cytology, and bacterial cultures in the CSF were all negative.

The EEG recorded one day before admission was unremarkable, without epileptiform discharges (Figure 1A); however, mild extreme delta brush, predominantly in the frontal regions, appeared on day 11 (Figure 1B) and became more prominent on day 17 (Figure 1C). Brain MRI obtained on day two with contrast was unremarkable, and pelvic MRI with contrast did not reveal an ovarian teratoma.

Antibody assay

Because of the suspicion of autoimmune encephalitis, three weeks after admission, CSF and serum obtained on day four of admission (before initiation of immunotherapy; pretreatment CSF/serum) were sent to the laboratory of Josep Dalmau (Dalmau’s Lab, Institut d’Investigació Biomèdica August Pi i Sunyer (IDIBAPS) Hospital Clinic, Barcelona, Spain), where neuronal surface (NS) antibodies were examined with established in-house cell-based assay (CBA) and rat brain tissue-based assay (TBA). NS antibodies examined included those against the NMDAR (GluN1), α-amino-3-hydroxy-5-methyl-4-isoxazolepropionic acid receptor (AMPAR), GABAA receptor (GABAAR), γ-aminobutyric acid B receptor (GABABR), metabotropic glutamate receptor 5 (mGluR5), metabotropic glutamate receptor 1 (mGluR1), dipeptidyl peptidase-like protein 6 (DPPX), contactin-associated protein-like 2 (Caspr2), leucine-rich glioma-inactivated 1 (LGI1), and neurexin 3. Among those, only GluN1 antibodies were found to be positive, confirming the diagnosis of anti-NMDAR encephalitis on day 39.

**Figure 2:**
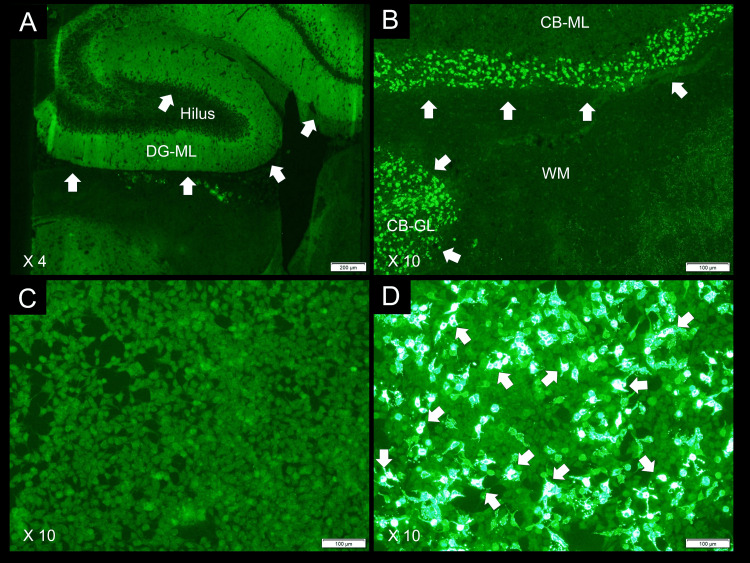
Tissue-based assay and fixed cell-based assay The arrows indicate intense homogeneous immunostaining on the DG-ML (A) and dot-like immunostaining on the CB-GL (B), consistent with NMDAR reactivity. The arrows in (D) highlight intense reactivity on GluN1 subunit-transfected cells, whereas no immunoreactivity was observed on control-transfected cells (C), confirming the presence of autoantibodies against NMDAR. These studies were performed using the patient’s CSF (diluted 1:2) with a kit (Euroimmun AG, Lübeck, Germany; product No: FA 111m-3) according to the manufacturer’s instructions, employing an indirect immunofluorescence assay. CBA, cell-based assay; DG-ML, dentate gyrus molecular layer; CB-ML, cerebellar molecular layer; CB-GL, cerebellar granular layer; WM, white matter; NMDAR, N-methyl-D-aspartate receptor

GluN1 antibody positivity of the pretreatment CSF obtained on day four was also subsequently examined at Kitasato University to see neuronal reactivity consistent with NMDAR [8] using a commercial kit (Euroimmun AG, Lübeck, Germany; product No: FA 111m-3) with an indirect immunofluorescent assay (Figure 2A, 2B). Intense reactivity of GluN1 antibodies was shown on a fixed CBA (Figure 2C, 2D). In addition, antibody titers in the pretreatment CSF were determined to be 1:2028 using the same kit based on the H-intensity scale (HIS) score strategy, as previously reported elsewhere [9].

Clinical course

On admission, the patient was able to communicate but soon fell into a delirious state with disinhibited psychiatric symptoms such as screaming, frequently uttering phrases like "I'm scared," "Stop," and "Help me," suggesting the presence of hallucinations, along with recurrent episodes of involuntary shaking. On day three, urinary retention developed, and she became episodically unresponsive, incoherent, or kept talking to herself. On day four, sinus tachycardia (up to 160 beats per minute) intermittently developed, with frequent occurrence of trembling seizures. Follow-up CSF examination obtained on day four revealed 26 WBCs/µL and a protein level of 27 mg/dL; HSV-PCR was again negative.

Given elevated anti-thyroid antibodies, Hashimoto’s encephalopathy was initially suspected, and intravenous high-dose methylprednisolone (IVMP, 1000 mg/day for three days) was started from day four. However, on day five, she fell into a less responsive state. She was treated with intravenous immunoglobulins (IVIg, 0.4 g/kg/day for five days) from day seven. On day eight, she developed generalized seizures with eye deviation to the right, for which continuous infusion of propofol (0.3 mg/kg/h) was started and subsequently titrated upward, and anti-seizure drugs were administered, beginning with levetiracetam at a dose of 1000 mg/day, which was subsequently increased to 3000 mg/day. Additionally, a loading dose of fosphenytoin (22.5 mg/kg) was administered intravenously. Propofol was tapered on day 14 and replaced with midazolam (0.1 mg/kg/h), which was initiated and titrated upward. Perampanel was also added at a dose of 2 mg/day from day 22. However, she developed central hypoventilation, ultimately leading to mechanical ventilation support on day 23.

At that point, anti-NMDAR encephalitis was highly clinically suspected. Accordingly, the patient was treated with three cycles of three-day IVMP, three cycles of IVIg, and one round of plasma exchange (PLEX, 350-400 mL/kg per exchange) administered twice a week for a total of four exchanges; however, no clinical improvement was observed. The patient was also treated with propofol, midazolam, levetiracetam (at up to 3000 mg/day), perampanel (at up to 8 mg/day), clonazepam (at up to 6 mg/day), and intravenous phenobarbital (used during severe episodes of status epilepticus). While waiting for the antibody test results from Dalmau’s Lab, the first dose of monthly intravenous cyclophosphamide (IVCPA, 750 mg/m²) was administered on day 35 [5]. Four days later, on day 39, the test results came back positive, confirming the diagnosis of anti-NMDAR encephalitis. The patient completed a total of four cycles of monthly IVCPA (750 mg/m²). During the treatment with IVCPA, leuprorelin (1.88 mg) was administered monthly to prevent ovarian toxicity.

Following the combined treatments with IVMP, IVIg, PLEX, and IVCPA, her condition gradually improved, allowing weaning from the ventilator, and she became able to express pain or thirst by the third month of hospitalization. By the fourth month of hospitalization, seizures resolved, and she became able to gargle or eat with assistance. Azathioprine (100 mg) was added from day 158. Six months after admission, the patient was transferred to a rehabilitation facility. On discharge, the modified Rankin Scale (mRS) score was 4, and she was prescribed azathioprine, levetiracetam, and anxiolytic medication for disinhibited irritability. One year after the onset of symptoms, the mRS score was 3. After that, her symptoms gradually improved, and she returned to her work. All medications were gradually tapered off. Four years after the onset of symptoms, the mRS score was 1.

## Discussion

The patient ultimately developed a typical spectrum of anti-NMDAR encephalitis, which fulfills the diagnostic criteria for “probable anti-NMDAR encephalitis” [4]. It is currently not difficult to make the clinical diagnosis with a high probability based on the clinical phenotypes; however, at presentation, when the patient was brought to the outpatient clinic one day before admission, she exhibited unusual trembling seizures consistent with a diagnosis of PNES, leading to a misdiagnosis of somatic symptom disorder. Accordingly, the clinical presentation of this case highlights that a patient with anti-NMDAR encephalitis can present with movement disorders or seizures consistent with PNES at the early stage of the disease before the development of major domains of symptoms, such as psychobehavioral or memory alterations, speech dysfunction, profoundly decreased level of consciousness, orofacial-limb dyskinesias, or epileptic seizures.

However, the subsequent clinical course of the disease was consistent with that of a severe case of anti-NMDAR encephalitis, the severity of which is usually associated with high antibody titers in CSF [9], increased intrathecal synthesis of GluN1 antibodies [10], or increased CSF CXCL13 concentration [11]. In general, first-line immunotherapies, including IVMP, IVIg, or PLEX, are ineffective in approximately half of anti-NMDAR encephalitis cases [5]. Among these refractory cases, those who received second-line immunotherapy, such as IVCPA and/or rituximab, achieve better functional outcomes than those who did not [5]. In our case, first-line immunotherapy had limited efficacy; therefore, IVCPA was chosen to improve prognosis, and four cycles were administered until clinical improvement was achieved. As a result, the patient improved to an mRS score of 1. Rituximab may be an ideal choice of second-line immunotherapy for refractory cases, but its off-label use is difficult in clinical practice in Japan, so we did not use it.

The patient’s course of anti-NMDAR encephalitis followed the typical progression of clinical phases. The systemic phase began with a transient fever. The psychiatric phase emerged with PNES-like episodes, agitation, hallucinations, and screaming episodes. During the neurologic phase, the patient experienced seizures, facial dyskinesia, postural abnormalities, and dysautonomia, including excessive sweating, urinary retention and central hypoventilation. Finally, in the recovery phase, after receiving second-line therapy with IVCPA, the patient gradually regained consciousness, achieved seizure control, and regained functional independence [1].

During the active stage, a high dose of anti-seizure medications, including anesthetic agents as well as levetiracetam, perampanel, and clonazepam, was required to suppress symptomatic seizures or refractory dyskinesias; however, no chronic use of anti-seizure medications is usually recommended in this disorder [12], so all medications, including anti-seizure medications, were gradually tapered off without relapse of epileptic seizures.

It is important that early symptoms of this disorder can mimic schizophrenia or somatic symptom disorder [1,13,14]. This patient presented to our hospital with trembling seizures mimicking PNES, which are often caused by psychological factors and frequently linked to stress or secondary gain [15]. As a result, the patient was initially suspected of having a somatic symptom disorder. However, her family discreetly observed her and confirmed that the same symptoms developed even when no one was visibly present, which did not support the hypothesis of secondary gain. Additionally, there was mild CSF pleocytosis with CSF-restricted OCB detection. These points differentiated PNES-mimicking seizures from true PNES. The PNES-mimicking episodes were later thought as early signs of anti-NMDAR encephalitis. Seizure-like episodes or paroxysmal movement disorders mimicking epileptic seizures without relevant epileptiform activity may not be rare in patients with anti-NMDAR encephalitis [16], but there have been no PNES-mimicking episodes captured on video. Careful observation of such movement disorders or seizures and early intervention with immunotherapies are essential to improve functional outcomes in patients with anti-NMDAR encephalitis.

## Conclusions

A patient with anti-NMDAR encephalitis can present with seizures closely resembling PNES, leading to a potential misdiagnosis and delayed initiation of treatment. An EEG recorded at an early stage, even when the patient exhibits PNES-mimicking episodes, can be normal, further increasing the risk of making a diagnosis of somatic symptom disorder. However, in this patient, EEG later revealed the characteristic pattern of extreme delta brush, and she subsequently followed a typical course of anti-NMDAR encephalitis, showing improvement following second-line therapy. Taking the time for a careful evaluation of atypical clinical features and making a diagnosis based on two established, independent assays performed in a research laboratory are essential to avoiding misdiagnosis/misconceptions and to ensuring timely immunotherapy.
